# Hypoxia-inducible factor-1α gene polymorphisms and cancer risk: a meta-analysis

**DOI:** 10.1186/1756-9966-28-159

**Published:** 2009-12-27

**Authors:** Tongfeng Zhao, Jing Lv, Jiangpei Zhao, Marius Nzekebaloudou

**Affiliations:** 1 Department of Geriatrics, the Second Affiliated Hospital, School of Medicine, Zhejiang University, 310009 Hangzhou, China; 2 Department of Geriatrics, Hangzhou Hospital of Traditional Chinese Medicine, Hangzhou, China; 3 Department of Food Science Nutrition, Zhejiang University, Hangzhou, China

## Abstract

**Background:**

The results from the published studies on the association between *hypoxia-inducible factor -1α *(HIF-1α) polymorphisms and cancer risk are conflicting. In this meta-analysis, we aimed to investigate the association between *HIF-1α *1772 C/T and 1790 G/A polymorphisms and cancer.

**Methods:**

The meta-analysis for 1772 C/T polymorphism included 4131 cancer cases and 5387 controls, and for 1790 G/A polymorphism included 2058 cancer cases and 3026 controls. Allelic and genotypic comparisons between cases and controls were evaluated. Subgroup analyses by cancer types, ethnicity, and gender were also performed. We included prostate cancer in male subgroup, and female specific cancers in female subgroup.

**Results:**

For the 1772 C/T polymorphism, the analysis showed that the T allele and genotype TT were significantly associated with higher cancer risk: odds ratio (OR) = 1.29 [95% confidence interval (CI, 1.01, 1.65)], P = 0.04, P_heterogeneity _< 0.00001, and OR = 2.18 [95% CI (1.32, 3.62)], P = 0.003, P_heterogeneity _= 0.02, respectively. The effect of the genotype TT on cancer especially exists in Caucasians and female subjects: OR = 2.40 [95% CI (1.26, 4.59)], P = 0.008, P_heterogeneity _= 0.02, and OR = 3.60 [95% CI (1.17, 11.11)], P = 0.03, P_heterogeneity _= 0.02, respectively. For the 1790 G/A polymorphism, the pooled ORs for allelic frequency comparison and dominant model comparison suggested a significant association of 1790 G/A polymorphism with a decreased breast cancer risk: OR = 0.28 [95% CI (0.08, 0.90)], P = 0.03, P_heterogeneity _= 0.45, and OR = 0.29 [95% CI (0.09, 0.97)], P = 0.04, P_heterogeneity _= 0.41, respectively. The frequency of the *HIF-1α *1790 A allele was very low and only two studies were included in the breast cancer subgroup.

**Conclusions:**

Our meta-analysis suggests that the *HIF-1α *1772 C/T polymorphism is significantly associated with higher cancer risk, and 1790 G/A polymorphism is significantly associated with decreased breast cancer risk. The effect of the 1772 C/T polymorphism on cancer especially exists in Caucasians and female subjects. Only female specific cancers were included in female subgroup, which indicates that the 1772 C/T polymorphism is significantly associated with an increased risk for female specific cancers. The association between the 1790 G/A polymorphism and lower breast cancer risk could be due to chance.

## Background

Cancer is one of the leading causes of death in the world. It has become a worldwide public health problem [1]. The exact mechanism of carcinogenesis is not yet fully elucidated [2]. Recently, it has become clear that genetic variation contributes to the development and progression of cancer [2,3]. However, due to various reasons, including considerable heterogeneity of the disease, the identification of susceptibility genes is difficult and most associations have not been replicated.

Intratumoral hypoxia is a hallmark of solid cancer [4]. A hypoxic microenvironment initiates multiple cellular responses, such as proliferation and angiogenesis, resulting in the development and progression of cancer [4]. Hypoxia-inducible factor -1 (HIF-1) is a key transcription factor that regulates cellular response to hypoxia [5,6]. Studies have demonstrated that HIF-1 plays important roles in the development and progression of cancer through activation of various genes that are involved in crucial aspects of cancer biology, including angiogenesis, energy metabolism, vasomotor function, erythropoiesis, and cell survival [5,6]. HIF-1 is a heterodimeric transcription factor consisting of α and β subunits [5,6]. The β subunit is constitutively expressed and the α subunit which determines HIF-1 activity is regulated by oxygen tension. Hypoxia- inducible factor -1α (HIF-1α) is hydroxylated and degraded rapidly under normoxic conditions through von Hippel-Lindau mediated ubiquitin-proteasome pathway whereas it becomes stabilized and is rapidly accumulated in cell under hypoxic conditions [5,6]. Recent studies have shown overexpression of HIF-1α in many human cancers with an advanced tumor grade, implying HIF-1α as an independent prognostic factor of cancer [7].

*HIF-1α *gene polymorphisms have been investigated for a possible role in mediating genetic predisposition to cancer [8]. Recently, two single nucleotide polymorphisms (SNPs) of human *HIF-1α *gene, *HIF-1α *1772 C/T (rs11549465) and 1790 G/A (rs rs11549467), which result in proline to serine and alanine to threonine amino acid substitutions, respectively, were identified. Both of them are located within exon 12 of the *HIF-1α *gene [5,6]. The presences of these polymorphic variants were shown to cause a significantly higher transcriptional activity than the activity of the wild type in vitro studies under both normoxic and hypoxic conditions [5,6]. Moreover, both of the polymorphisms were associated with increased tumor microvessel density, thus contributing to the development and progression of cancer [5,6]. A number of investigators have studied the possible association between the *HIF-1α *polymorphisms and cancer risk, but the results have been conflicting [5,6,8-22]. Thus, the association between the *HIF-1α *1772 C/T and 1790 G/A polymorphisms and cancer requires further investigation. In this paper, a meta-analysis was performed on previous reports to investigate the association of *HIF-1α *1772 C/T and 1790 G/A polymorphism with cancer.

## Materials and methods

### Identification and eligibility of relevant studies

All studied published before June 2009 that investigated the association between the *HIF-1α *1772 C/T and 1790 G/A polymorphisms with cancer were considered in the meta-analysis. A systematic search of the literature was carried out by using PubMed. The language was limited to English. The keywords used for this search were "HIF-1 OR hypoxia-inducible factor-1" concatenated with "polymorphism OR variant OR SNP OR mutation" AND "cancer OR tumor OR carcinoma OR malignancy". Only the studies with complete data on comparison of frequency of the *HIF-1α *1772 C/T and 1790 G/A gene polymorphisms between controls and patients with cancer were selected. Animal studies, case reports, review articles, abstracts, editorials, reports with incomplete data, and studies based on pedigree data were excluded.

### Data extraction

Two investigators independently reviewed the articles to exclude irrelevant and overlapping studies. The results were compared, and disagreements were resolved by discussion and consensus. When overlapping articles were found, we only included the publication that reported the most extensive information. From each study, the following information was extracted: journal, year of publication, first author, demographics, racial background of the study population, validity of the genotyping method, matching, and the number of cases and controls for each genotype. Frequencies of alleles were calculated for the cases and the controls, from the corresponding genotype distributions.

### Statistical analysis

Review Manager 5.0 software (The Cochrane Collaboration, Oxford, UK) was used for meta-analysis. The following genotype contrasts for the *HIF-1α *1772 C/T polymorphism were evaluated: homozygotes TT versus a combination of CT and CC [TT versus (CT+CC), recessive model], a combination of TT and CT versus CC [(TT+CT) versus CC, dominant model]. Contrast of C allelic frequency versus G allelic frequency (C versus G) was also evaluated. A allele of the *HIF-1α *1790 G/A polymorphism was very rare. In most of the studies, homozygote AA was totally absent in both case and controls. For the *HIF-1α *1790 G/A polymorphism, we only performed allelic frequency comparison (A versus G) and dominant model comparison [(AA+AG) versus GG]. In addition, we conducted subgroup analyses by cancer types, ethnicity, and gender. For gender subgroups, we included prostate cancer in male subgroup, and female specific cancers such as breast cancer, endometrial cancer, ovarian cancer and cervix cancer in female subgroup. We only conducted the meta-analysis on the subgroup with more than two studies in Hardy-Weinberg equilibrium (HWE). For the *HIF-1α *1790 G/A polymorphism, the pooled effects for other cancers (exclusion of the study on breast cancer) were also performed.

The existence of heterogeneity between studies was ascertained by Q-statistic. The pooled odds ratio (OR) was estimated with models based on fixed effects or random effects assumptions. If the significant Q statistic (P < 0.1) indicated heterogeneity across studies, a random effects model was used for meta-analysis. Otherwise, a fixed effect model was selected. The 95% confidence interval (CI) of OR was also calculated. The distributions of genotypes in the controls were checked for HWE. Studies with the controls not in HWE were subjected to a sensitivity analysis [23].

The publication bias among the studies from the cases versus controls was assayed. Funnel plots of the *HIF-1α *1772 C/T polymorphism for T versus C and *HIF-1α *1790 G/A polymorphism for A versus G were performed to look for evidence of publication bias. The funnel plot should be asymmetric when there is publication bias and symmetric in the case of no publication bias. Egger's test, estimated by MIX 1.7 software (Kitasato Clinical Research Center, Kitasato University, Japan), was performed to measure the funnel plot asymmetry [24-26].

## Results

### Eligible studies

The flow diagram illustrates the main reasons for studies exclusion (Additional file 1). The selected study characteristics were summarized in Additional file 2. 16 relevant case-control studies concerning the *HIF-1α *1790 G/A and 1772 C/T polymorphisms and cancer were included in the meta-analysis. In all 16 studies, there were 9 studies of Caucasians, 5 studies of East Asians, 2 studies of mixed ethnicity. The 16 studies included 4 studies on prostate cancer, 3 studies on breast cancer, 2 studies on colorectal carcinoma, 2 studies on renal cell carcinoma, 1 studies on endometrial cancer, 1 study on early stage of oral squamous cell carcinoma, 1 study on ovarian cancer, endometrial cancer, and cervical cancer, 1 study on esophageal squamous cell carcinoma, and 1 study on head and neck squamous cell carcinoma. The samples only consisted of females in 7 studies, only consisted of males in 4 studies, and consisted of both females and males in 5 studies. In the eligible studies, all the 16 studies presented the data on the 1772 C/T polymorphism, 10 studies presented the data on the 1790 G/A polymorphism. For the 1772 C/T polymorphism, the distributions of the genotypes in the control groups in 5 studies were not in HWE. For the 1790 G/A polymorphism, the distributions of the genotypes in control groups in 1 study were not in HWE. In all the eligible studies, 1 study provided data on three kinds of cancers (endometrial cancer, ovarian cancer, and cervical cancer) and both of the polymorphisms. Thus, each type of cancer in the study was treated as a separate study in this meta-analysis. In the eligible studies, 7 studies stated that the age, gender status or other variables were matched between the cases and controls, 1 paper just stated the controls were matched within constraints and did not describe the variables in detail, and 8 studies did not clearly state the use of a matching design for cases during the selection process of controls. Genotyping methods used in the eligible studies included PCR-restriction fragment length polymorphism (PCR-RFLP), direct sequencing, PCR-single strand conformational polymorphism (PCR-SSCP), and SNP-IT™ assays. Only 11 studies mentioned quality control of the genotyping, such as blindness to the case-control status, random repeat, or validation using a different genotyping method. The genotype and allele distribution of the *HIF-1α *1772 C/T and 1790 G/A polymorphisms of individual studies were summarized in Additional file 3.

### Summary statistics

The meta-analysis for the *HIF-1α *1772 C/T polymorphism included 4131 cancer cases and 5387 controls. In both case group and control group, allele C was the most frequent, and the prevalence of the CC genotype was the highest, whilst the prevalence of the TT genotype was the lowest (Additional file 2, 3).

The meta-analysis for the *HIF-1α *1790 G/A polymorphism included 2058 cancer cases and 3026 controls. In both case group and control group, allele G was the most frequent, and the prevalence of the GG genotype was the highest, whilst the prevalence of the AA genotype was the lowest (Additional file 2, 3).

### Association of the HIF-1α 1772 C/T polymorphism with cancer risk

**Figure 1:**
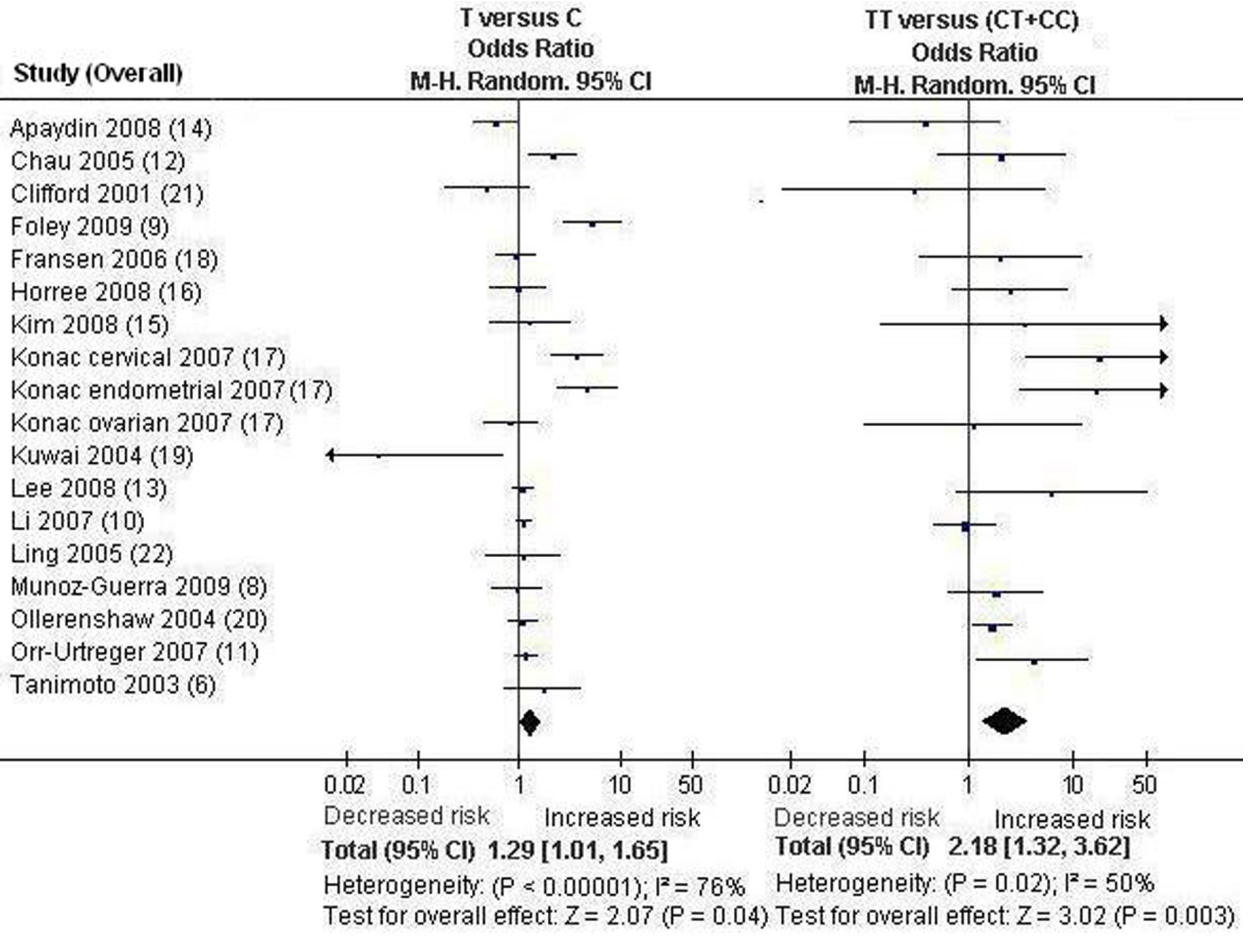
**Forest plot of the *HIF-1α *1772 C/T polymorphism and cancer risk [T versue C and TT versus (CT+CC)]**. Results from the analysis on all available studies.

**Figure 2:**
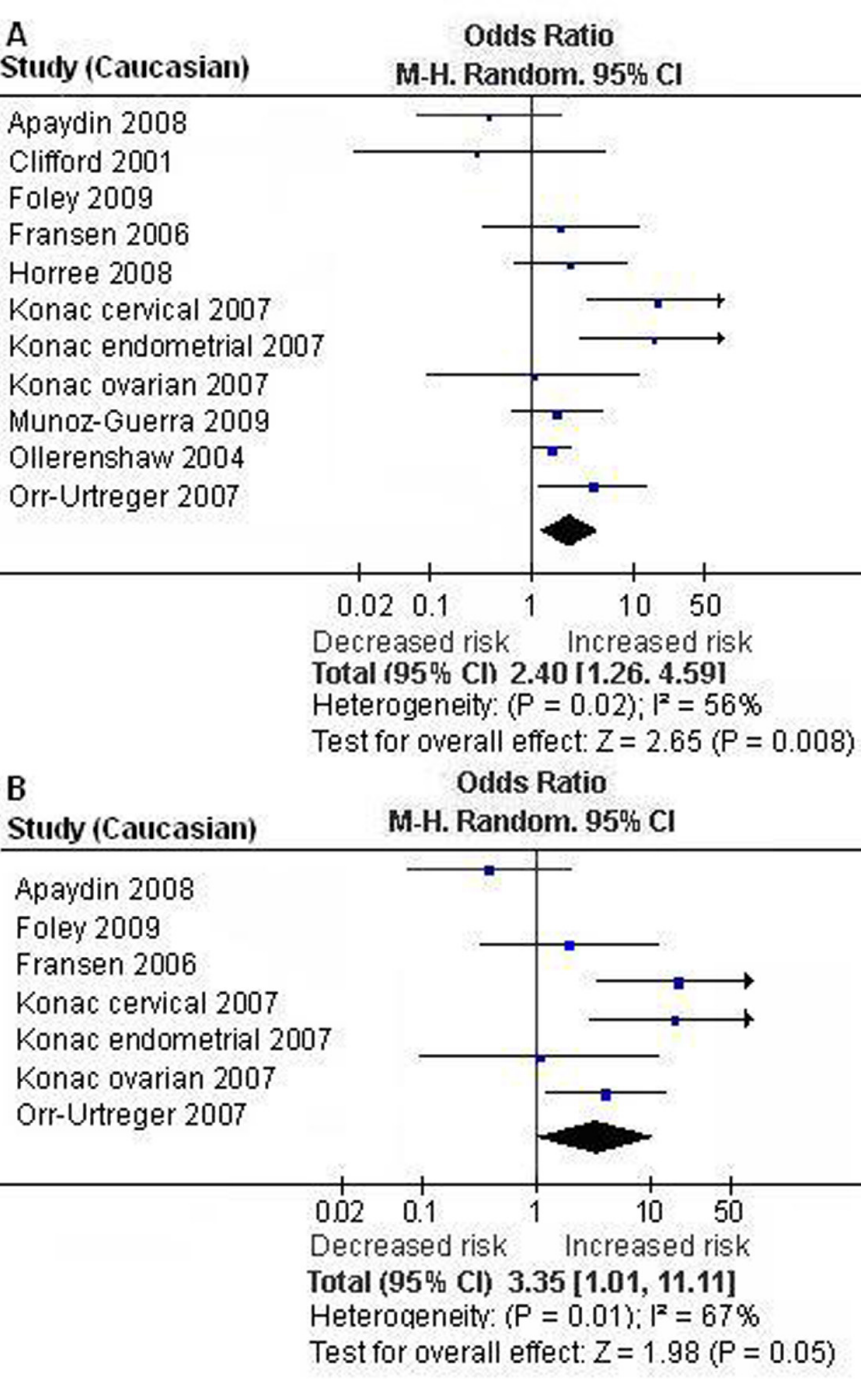
**Forest plot the *HIF-1α *1772 C/T polymorphism and cancer risk in Caucasians [TT versus (CT+CC)]**. A. Results from the analysis on all studies of Caucasians. B. Results from the sensitivity analysis (exclusion of the studies with controls not in Hardy-Weinberg equilibrium).

**Figure 3:**
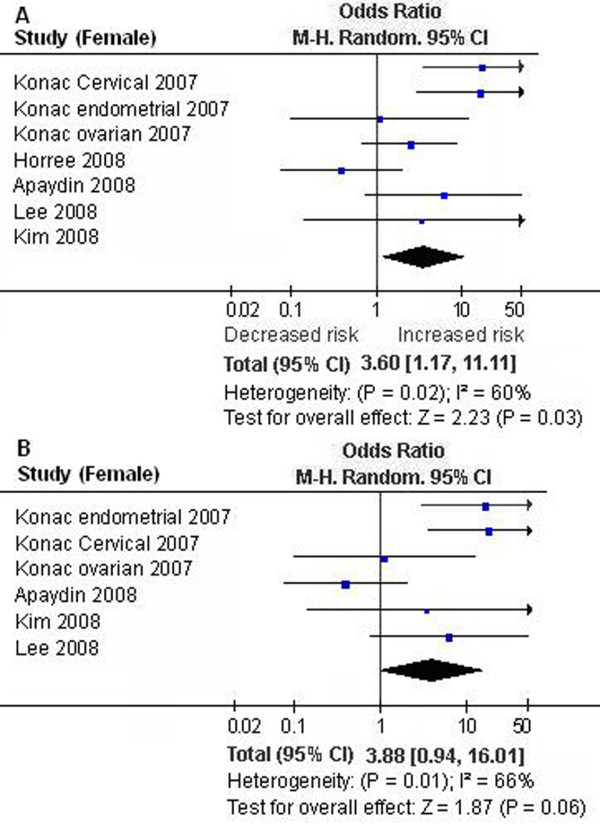
**Forest plot the *HIF-1α *1772 C/T polymorphism and cancer risk in female subjects [TT versus (CT+CC)]**. A. Results from the analysis on all studies of female subjects. B. Results from the sensitivity analysis (exclusion of the studies with controls not in Hardy-Weinberg equilibrium).

We first performed the meta-analysis on all 18 studies. The pooled ORs for allelic frequency comparison and recessive model comparison suggested that the T allele and genotype TT were significantly associated with an increased cancer risk: OR = 1.29 [95% CI (1.01, 1.65)], P = 0.04, P_heterogeneity _< 0.00001, and OR = 2.18 [95% CI (1.32, 3.62)], P = 0.003, P_heterogeneity _= 0.02, respectively (Table 1, Figure 1). We then performed the subgroup analyses stratified by cancer types, ethnicity and gender. The pooled ORs for allelic frequency comparison and dominant model comparison suggested the 1772 C/T polymorphism was significantly associated with an increased prostate cancer risk: OR = 1.78 [95% CI (1.07, 2.94)], P = 0.03, P_heterogeneity _< 0.0001, and OR = 1.85 [95% CI (1.04, 3.31)], P = 0.04, P_heterogeneity _< 0.0001, respectively (Table 1). The association between the genotype TT and increased cancer susceptibility was significant in Caucasians and in female subjects: OR = 2.40 [95% CI (1.26, 4.59)], P = 0.008, P_heterogeneity _= 0.02, and OR = 3.60 [95% CI (1.17, 11.11)], P = 0.03, P_heterogeneity _= 0.02 (Table 1, Figure 2, 3). A marginal significant association between the 1772 C/T polymorphism and increased cancer risk was detected in East Asians under recessive model: OR = 5.31 [95% CI (0.91, 30.83)], P = 0.06, P_heterogeneity _= 0.76 (Table 1). The remaining pooled ORs from this analysis were not significant (P > 0.05) (Table 1).

**Table 1:** Meta-analysis of the HIF-1α 1772 C/T polymorphism and cancer association.

Genetic contrasts	Group and subgroups under analysis	Studies (n)	Q test *P *value	Model seclected	OR (95% CI)	*P*
T versus C	Overall	18	<0.00001	Random	1.29 (1.01, 1.65)	0.04
	Overall in HWE	13	<0.00001	Random	1.39 (1.02, 1.90)	0.04
	Caucasian	11	<0.00001	Random	1.33 (0.90, 1.97)	0.15
	Caucasian in HWE	7	<0.00001	Random	1.69 (0.94, 3.04)	0.08
	East Asian	5	0.16	Fixed	1.05 (0.84, 1.30)	0.69
	Female*	7	<0.00001	Random	1.39 (0.83, 2.35)	0.21
	Female in HWE*	6	<0.00001	Random	1.48 (0.81, 2.71)	0.20
	Male (prostate cancer)**	4	<0.0001	Random	1.78 (1.07, 2.94)	0.03
	Male (prostate cancer) in HWE**	3	<0.0001	Random	1.68 (0.94, 3.02)	0.08
	Breast cancer	3	0.12	Fixed	0.99 (0.79, 1.23)	0.90
	Colorectal cancer	2	0.02	Random	0.26 (0.01, 6.38)	0.41
TT versus (CT+CC)	Overall	18	0.02	Random	2.18 (1.32, 3.62)	0.003
	Overall in HWE	13	0.002	Random	2.87 (1.14, 7.26)	0.03
	Caucasian	11	0.02	Random	2.40 (1.26, 4.59)	0.008
	Caucasian in HWE	7	0.01	Random	3.35 (1.01, 11.11)	0.05
	East Asian	5	0.76	Fixed	5.31 (0.91, 30.83)	0.06
	Female*	7	0.02	Random	3.60 (1.17, 11.11)	0.03
	Female in HWE*	6	0.01	Random	3.88 (0.94, 16.01)	0.06
	Male (prostate cancer)**	4	0.1	Fixed	1.53 (0.90, 2.60)	0.11
	Male (prostate cancer) in HWE**	3	0.04	Random	1.78 (0.41, 7.74)	0.44
	Breast cancer	3	0.10	Fixed	1.51 (0.55, 4.11)	0.42
	Colorectal cancer	2	-	Random	1.97 (0.33, 11.90)	0.46
(TT+CT) versus CC	Overall	18	<0.00001	Random	1.19 (0.88, 1.59)	0.26
	Overall in HWE	13	<0.00001	Random	1.34 (0.97, 1.85)	0.08
	Caucasian	11	<0.00001	Random	1.15 (0.68, 1.93)	0.61
	Caucasian in HWE	7	<0.00001	Random	1.70 (0.89, 3.26)	0.11
	East Asian	5	0.15	Fixed	1.01 (0.80, 1.27)	0.96
	Female*	7	0.0004	Random	1.28 (0.76, 2.15)	0.35
	Female in HWE*	6	0.0002	Random	1.41 (0.77, 2.57)	0.26
	Male (prostate cancer)**	4	<0.0001	Random	1.85 (1.04, 3.31)	0.04
	Male (prostate cancer) in HWE**	3	<0.0001	Random	1.75 (0.89, 3.47)	0.11
	Breast cancer	3	0.22	Fixed	0.96 (0.76, 1.21)	0.75
	Colorectal cancer	2	0.02	Random	0.25 (0.01, 5.99)	0.39

OR, odds ratio; CI, confidence interval; HWE, Hardy-Weinberg equilibrium.

* Only female specific cancers were included in the female subgroup.

** All male patients were the patients with prostate cancer.

Sensitivity analysis was next performed by excluding the studies with controls not in HWE. The results from the allelic frequency comparison and dominant model comparison showed no evidence that the 1772 C/T polymorphism was significantly associated with an increased prostate cancer risk: OR = 1.68 [95% CI (0.94, 3.02)], P = 0.08, P_heterogeneity _< 0.0001, and OR = 1.75 [95% CI (0.89, 3.47)], P = 0.11, P_heterogeneity _< 0.0001, respectively (Table 1). The association between the genotype TT and the increased cancer risk was marginally significant in Caucasians and in female subjects: OR = 3.35 [95% CI (1.01, 11.11)], P = 0.05, P_heterogeneity _= 0.01, and OR = 3.88 [95% CI (0.94, 16.01)], P = 0.06, P_heterogeneity _= 0.01, respectively (Table 1, Figure 2, 3). The other results were similar to those when the studies with controls not in HWE were included (Table 1).

There was significant heterogeneity among the available studies (Table 1). To detect the source of the heterogeneity, we performed the subgroup analyses by gender, cancer types, and ethnicity. The results showed studies in female subject subgroup and Caucasian subgroup were the main contributors of heterogeneity (Table 1).

### Association of the HIF-1α 1790 G/A polymorphism with cancer risk

**Figure 4:**
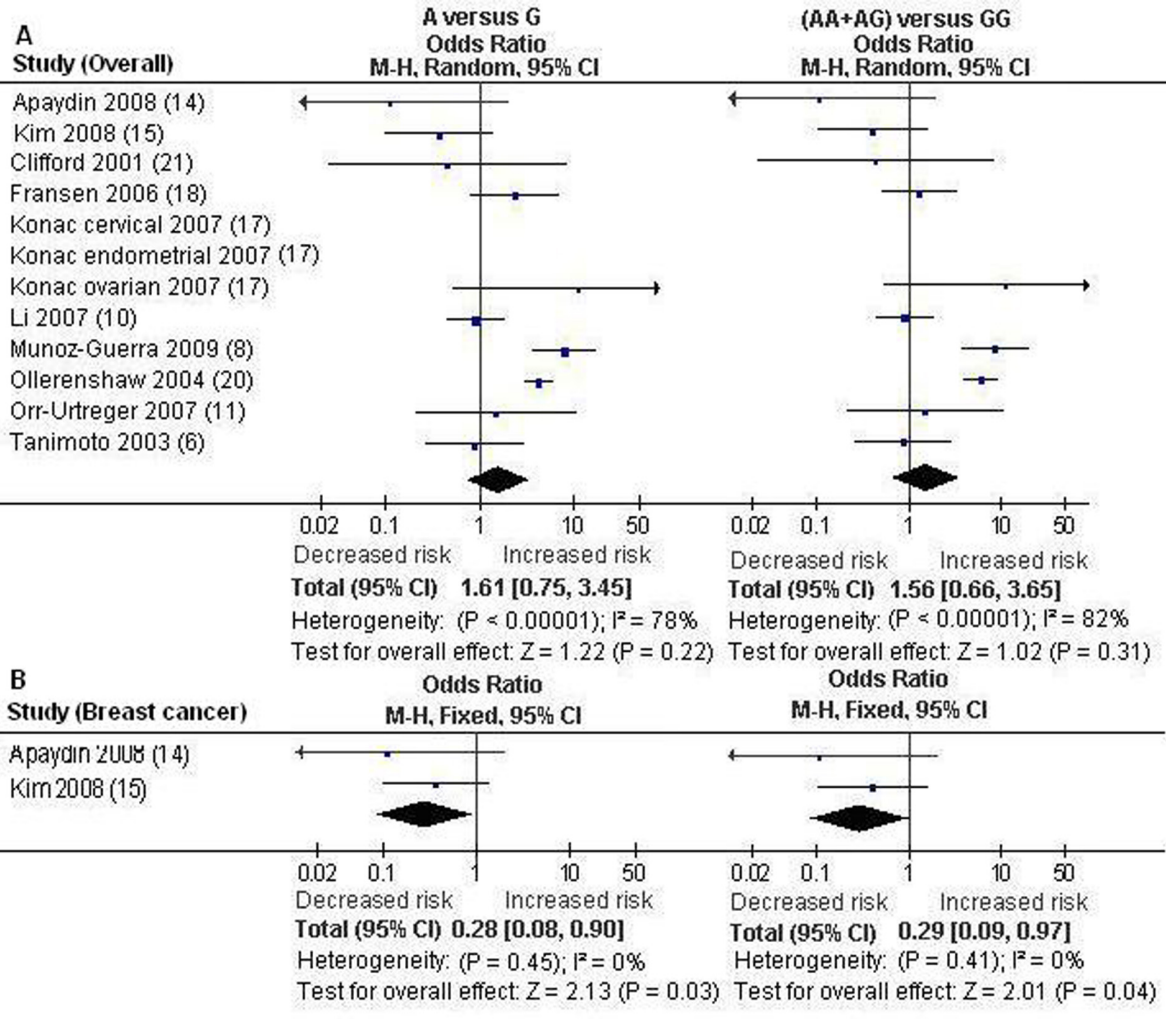
**Forest plot the *HIF-1α *1790 G/A polymorphism and cancer risk [A versue G and (AA+AG) versus GG]**. A. Results from the analysis on all available studies. B. Results from the analysis on breast cancer subgroup.

The results on all 12 studies showed no evidence that the *HIF-1α *1790 G/A polymorphism was significantly associated with an increased cancer risk (P > 0.05) (Table 2, Figure 4). The significant association between the A allele and the increased cancer risk was detected in other cancers: OR = 2.31 [95% CI (1.12, 4.75)], P = 0.02, P_heterogeneity _= 0.0004 (Table IV) (Table 2). A marginal association between the 1790 G/A polymorphism and the increased cancer risk in other cancers was also detected under dominant model: OR = 2.22 [95% CI (0.95, 5.20)], P = 0.06, P_heterogeneity _< 0.00001 (Table 2). The pooled ORs for allelic frequency comparison and dominant model comparison suggested the 1790 G/A polymorphism was significantly associated with an increased cancer risk in Caucasians: OR = 3.08 [95% CI (1.49, 6.36)], P = 0.002, P_heterogeneity _= 0.04, and OR = 2.60 [95% CI (1.03, 6.59)], P = 0.04, P_heterogeneity _= 0.002, respectively (Table 2). However, reanalysis after exclusion the studies with controls not in HWE did not suggest these associations (P > 0.05) (Table 2). The pooled ORs for A versus G and (AA+AG) versus GG suggested that 1790 G/A polymorphism was significantly associated with a decreased breast cancer risk: OR = 0.28 [95% CI (0.08, 0.90)], P = 0.03, P_heterogeneity _= 0.45, and OR = 0.29 [95% CI (0.09, 0.97)], P = 0.04, P_heterogeneity _= 0.41, respectively (Table 2, Figure 4). The remaining pooled ORs on the association of 1790 G/A polymorphism and cancer risk were not significant (P > 0.05) (Table 2).

**Table 2:** Meta-analysis of the *HIF-1α *1790 G/A polymorphism and cancer association.

Genetic contrasts	Group and subgroups under analysis	Studies (n)	Q test *P* value	Model seclected	OR (95% CI)	*P*
A versus G	Overall	12	<0.00001	Random	1.61 (0.75, 3.45)	0.22
	Overall in HWE	11	0.0002	Random	1.32 (0.54, 3.24)	0.54
	Caucasian	9	0.04	Random	3.08 (1.49, 6.36)	0.002
	Caucasian in HWE	8	0.02	Random	2.15 (0.66, 7.02)	0.20
	East Asian	2	0.33	Fixed	0.58 (0.24, 1.40)	0.23
	Female*	5	0.07	Random	0.65 (0.07, 6.05)	0.71
	Male (prostate cancer)**	2	0.64	Fixed	0.96 (0.49, 1.90)	0.91
	Breast cancer	2	0.45	Fixed	0.28 (0.08,0.90)	0.03
	Other cancers	10	0.0004	Random	2.31 (1.12, 4.75)	0.02
	Other cancers in HWE	9	0.002	Random	1.97 (0.79, 4.90)	0.15
(AA+AG) versus GG	Overall	12	<0.00001	Random	1.56 (0.66, 3.65)	0.31
	Overall in HWE	11	0.0004	Random	1.25 (0.53, 2.97)	0.61
	Caucasian	9	0.002	Random	2.60 (1.03, 6.59)	0.04
	Caucasian in HWE	8	0.004	Random	1.80 (0.50, 6.54)	0.37
	East Asian	2	0.41	Fixed	0.61 (0.25, 1.51)	0.29
	Female*	5	0.08	Random	0.68 (0.07, 6.30)	0.74
	Male (prostate cancer) **	2	0.64	Fixed	0.96 (0.49, 1.90)	0.91
	Breast cancer	2	0.41	Fixed	0.29 (0.09, 0.97)	0.04
	Other cancers	10	<0.00001	Random	2.22 (0.95, 5.20)	0.06
	Other cancers in HWE	9	0.002	Random	1.78 (0.72, 4.43)	0.21

OR, odds ratio; CI, confidence interval; HWE, Hardy-Weinberg equilibrium.

* Only female specific cancers were included in the female subgroup.

** All male patients were the patients with prostate cancer

There was significant heterogeneity for allelic frequency comparison and dominant model comparison among the available studies (Table 2). However, the heterogeneity was effectively decreased or removed in the subgroups stratified by gender, ethnicity, and cancer types (Table 2).

### Publication bias

Publication bias was assayed by visual funnel plot inspection and Egger's test. The funnel plots for T versus C were basically symmetric (Additional file 4A) and Egger's test did not indicate asymmetry of the plot [Intercept = 0.5092, 95% CI (-1.5454, 2.5639), P = 0.6065]. The funnel plots for A versus G showed some asymmetry that could suggest the existence of publication bias (Additional file 4B). However, Egger's test did not show statistical evidence for publication bias [Intercept = -1.82, 95% CI (-4.1611, 0.5212), P = 0.1108].

## Discussion

HIF-1 plays a major role in cancer progression and metastasis through activation of various genes that are linked to regulation of angiogenesis, cell survival, and energy metabolism [5,6]. The *HIF-1α *gene was previously found to be implicated in the development and progression of cancer [5,6]. The polymorphisms analyzed in the present study consist of C to T and G to A nucleotide substitutions at positions 1772 and 1790 of the exon 12 of the *HIF-1α *gene [5,6]. Because a study by Tanimoto et al [6] showed that both of the substitutions displayed an increased transactivation capacity of HIF-1α in vitro, the presence of the variant alleles might be associated with increased cancer susceptibility. However, studies focusing on the association of the *HIF-1α *gene polymorphism with cancer susceptibility had controversial conclusions [5,6,8-22]. The lack of concordance across many of these studies reflects limitation in the studies, such as small sample sizes, ethnic difference and research methodology. Meta-analysis is a powerful tool for summarizing the results from different studies by producing a single estimate of the major effect with enhanced precision. It can overcome the problem of small sample size and inadequate statistical power of genetic studies of complex traits, and provide more reliable results than a single case-control study [27].

In this meta-analysis, we investigated the association between the *HIF-1α *1772 C/T and 1790 G/A polymorphism and cancer risk. The subgroup analyses stratified by cancer types, ethnicity, and gender were also performed. For the *HIF-1α *1772 C/T polymorphism, our meta-analysis on the available studies showed that the T allele and genotype TT were significantly associated with increased cancer risk. These associations were very robust, which did not vary materially when the sensitivity analyses (exclusion the study with controls not in HWE) were performed. The effect of the genotype TT on cancer especially exists in Caucasians and female subjects. Only female specific cancers were included in female subgroup in our meta-analysis, which indicates that the genotype TT is significantly associated with an increased risk for female specific cancers. The molecular basis of gender specific effect of the *HIF-1α *1772 C/T polymorphism on cancers is unclear. Studies have shown that estrogen can induce the expression of *HIF-1α *[28,29]. The substitution of C to T at positions 1772 of the exon 12 of the *HIF-1α *gene further increase the transactivation capacity of the *HIF-1α *gene and thus promote the development of female specific cancers. We also observed a marginally significant association between the genotype TT and increased cancer risk in East Asians. However, subjects with mutant homozygotes were only detected in two studies of East Asians. The CI for this subgroup was very wide, and the association could have been caused by chance. More studies based on larger population should be conducted to further examine this association.

For the *HIF-1α *1790 G/A polymorphism, the meta-analysis on all studies showed no evidence that the *HIF-1α *1790 G/A polymorphism was significantly associated with increased cancer risk. We also performed the stratification analyses by gender, ethnicity, and cancer types. The pooled ORs for allelic frequency comparison and dominant model comparison suggested the 1790 G/A polymorphism was significantly associated with an increased cancer risk in Caucasians. However, the sensitivity analysis did not suggest this association. Because the results from the sensitivity analysis were more valid, our meta-analysis does not strongly suggest the association between the *HIF-1α *1790 G/A polymorphism and cancer risk in Caucasians [23]. The pooled effects for allelic frequency comparison and dominant model comparison suggested a significant association between the *HIF-1α *1790 G/A polymorphism and a decreased breast cancer risk. Because the conclusion is inconsistent with the general understanding that the 1790 A alleles enhances *HIF-1α *transcriptional activity and the presence of the variant allele might be associated with increased cancer susceptibility, we further performed the meta-analysis for the other cancers to detect the specific effects of cancer type [6]. The results suggested a significant association between the A allele and increased cancer risk in other cancers. A marginal association between the 1790 G/A polymorphism and increased cancer risk in other cancers was also detected under dominant model. However, the reanalysis after exclusion the studies with controls not in HWE did not suggest these associations. Our meta-analysis does not strongly support the association between the *HIF-1α *1790 G/A polymorphism and the cancer risk in other cancers. The exact mechanism for the inverse association between the *HIF-1α *1790 G/A polymorphism and breast cancer was not clear. However, there were two factors that must be considered. First, the frequency of the *HIF-1α *1790 A allele was very low and only two studies were included in the breast cancer subgroup. So, the association could be due to chance. Second, our meta-analysis suggests that carcinogenic mechanism may differ in different cancers and *HIF-1α *1790 G/A polymorphism may exert varying effect. More studies will be required to further examine the association.

The current meta-analysis has several limitations which should be noted. First, the meta-analysis was based on the aggregation of published case-control studies. 8 studies did not clearly state the use of a matching design for cases during the selection process of controls. The meta-analysis was based on unadjusted estimates. A more precise analysis should be conducted if more detailed individual data were available, which would allow for an adjusted estimate. Second, because of data limitation, we did not perform the stratification analyses by age, smoking, or other variables. Third, several genotyping methods were used in the eligible studies. The quality control of genotyping was not well documented in some studies. Undoubtedly, the limitations mentioned should affect our final conclusions.

## Conclusions

Our meta-analysis suggests that the *HIF-1α *1772 C/T polymorphism is significantly associated with higher cancer risk, and the 1790 G/A polymorphism is significantly associated with decreased breast cancer risk. The effect of the 1772 C/T polymorphism on cancer especially exists in Caucasians and female subjects. Only female specific cancers were included in female subgroup, which indicates that the 1772 C/T polymorphism is significantly associated with an increased risk for female specific cancers. The association between the 1790 G/A polymorphism and lower breast cancer risk could be due to chance.

## Abbreviations

CI: confidence interval; HWE: Hardy-Weinberg equilibrium; HIF-1: hypoxia- inducible factor -1; HIF-1α: hypoxia- inducible factor -1α; OR: odds ratio; SNP: single nucleotide polymorphism.

## Competing interests

The authors declare that they have no competing interests.

## Authors' contributions

TFZ participated in the design, data acquisition, manuscript writing, and have given final approval of the version to be published. JPZ performed data analysis, data interpretation. JL participated in the design, data acquisition. MN participated in data analysis and drafting the manuscript. All authors read and approved the final manuscript.

## Supplementary Material

Additional file 1 The flow diagram of included/excluded studies. Click here for file

Additional file 2 Characteristics of individual studies included in the meta-analysis. Click here for file

Additional file 3 Genotype and allele distribution of *hypoxia- inducible factor -1α *1772 C/T and 1790 G/A polymorphisms of individual studies included in the meta-analysis. Click here for file

Additional file 4 **Funnel plots for publication bias test**. A. *HIF-1α *1772 C/T: T versus C. B. *HIF-1α *1790 G/A: A versus G. Each point represents a separate study for the indicated association. SE(SMD), standard error of the logarithm of the odd ratio. Click here for file
