# Supplementary figures and images for: Hypoxia-inducible factor-1α gene polymorphisms and cancer risk: a meta-analysis
Source: J Exp Clin Cancer Res. 2009 Dec 27;28(1):159. doi: 10.1186/1756-9966-28-159 (PMC2804603; doi:10.1186/1756-9966-28-159)

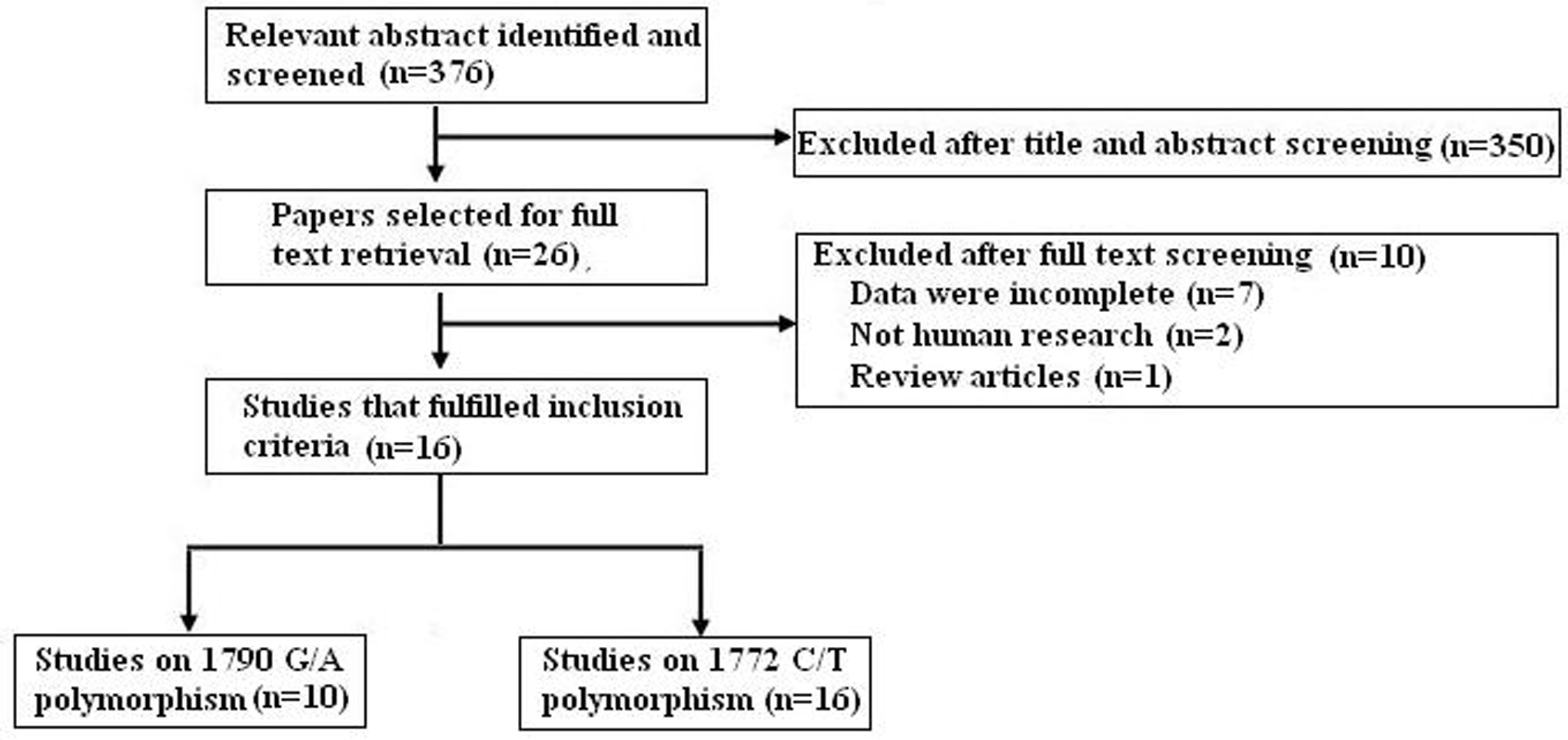

Supplement: Additional file 1 — The flow diagram of included/excluded studies. [file 1756-9966-28-159-S1.JPEG]

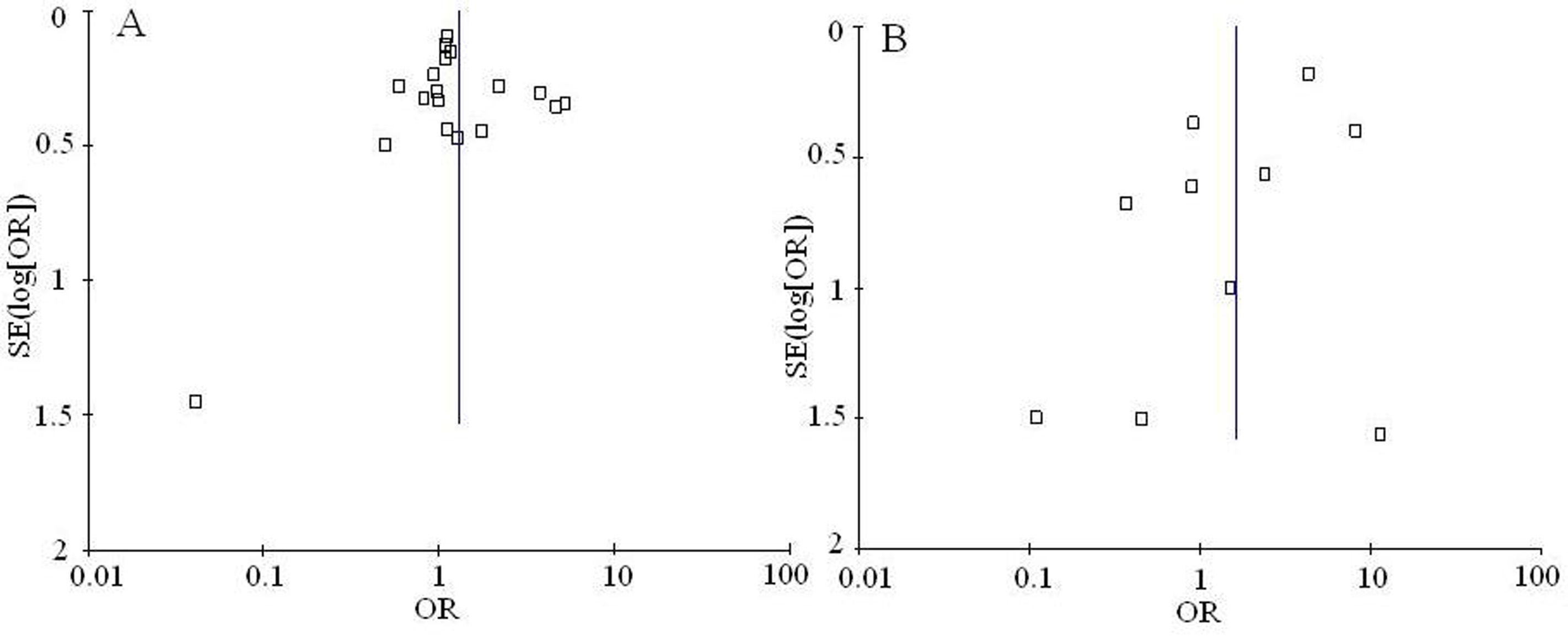

Supplement: Additional file 4 — Funnel plots for publication bias test. A. HIF-1α 1772 C/T: T versus C. B. HIF-1α 1790 G/A: A versus G. Each point represents a separate study for the indicated association. SE(SMD), standard error of the logarithm of the odd ratio. [file 1756-9966-28-159-S4.JPEG]
