# Supplementary material for: Hypoxia-inducible factor-1α gene polymorphisms and cancer risk: a meta-analysis
Source: J Exp Clin Cancer Res. 2009 Dec 27;28(1):159. doi: 10.1186/1756-9966-28-159 (PMC2804603; doi:10.1186/1756-9966-28-159)
Supplement: Additional file 2 — Characteristics of individual studies included in the meta-analysis. [file 1756-9966-28-159-S2.DOC]

**Additional file 2: Characteristics of individual studies included in the meta-analysis.**

| First author/Year | Country | Ethnicity | Cancer type | Gender | Number of  cases/controls | | Genotyping | HWE | | Matching variables |
| --- | --- | --- | --- | --- | --- | --- | --- | --- | --- | --- |
|  |  |  |  |  | 1790 G/A | 1772 C/T |  | 1790 G/A | 1772 C/T |  |
| Foley/2009 [9]  Li/2007 [10]  Orr-Urtreger/  2007 [11]  Chau/2005 [12]  Lee/2008 [13]  Apaydin/2008 [14]  Kim/2008 [15]  Horree/2008 [16]  Konac/2007 [17]  Fransen/2006 [18]  Kuwai/2004 [19]  Ollerenshaw/  2004 [20]  Clifford/2001 [21]  Munoz-Guerra/  2009 [8]  Ling/2005 [22]  Tanimoto/2003 [6] | Ireland  USA  Israel  USA  Korea  Turkey  Korea  Netherlands  Turkey  Sweden  Japan  UK  UK  Spain  China  Japan | Caucasian  Mixed  Caucasian  Mixed  East Asian  Caucasian  East Asian  Caucasian  Caucasian  Caucasian  Caucasian  Caucasian  East Asian  Caucasian  Caucasian  Caucasian  East Asian  East Asian | Prostate cancer  Prostate cancer  Prostate cancer  Prostate cancer  Breast cancer  Breast cancer  Breast cancer  Endometrial cancer  Endometrial cancer ^a^  Ovarian cancer  Cervical cancer ^a^  Colorectal carcinoma  Colorectal carcinoma  Renal cell carcinoma  Renal cell carcinoma  Oral squamous cell carcinoma  Esophageal squam-  ous cell carcinoma  Head and neck squ- amous cell carcinoma | M  M  M  M  F  F  F  F  F  F  F  M/F  M/F  M/F  M/F  M/F  M/F  M/F | -  1066/1264  200/300  -  -  102/102  90/102  -  21/107  49/107  32/107  198/256  -  146/288  35/144  64/139  -  55/110 | 95/188  1041/1234  402/300  196/196  1332/1369  102/102  90/102  58/559  21/107  49/107  32/107  198/258  100/100  160/162  35/143  70/139  95/104  55/110 | Direct sequencing  PCR-RFLP  PCR-RFLP  Direct sequencing  SNP-IT™ assays  PCR-RFLP, direct sequencing  Direct sequencing  Direct sequencing  PCR-RFLP  PCR-RFLP, PCR-SSCP  PCR-SSCP  PCR-RFLP  Direct sequencing  PCR-SSCP  PCR-RFLP  PCR-RFLP,  Direct sequencing  Direct sequencing | In  In  In  In  In  In  In  In  In  In  In  In  In  No  In  In  In  In | In  In  In  No  In  In  In  No  In  In  In  In  In  No  No  No  In  In | Age  Age, smoking status  NA  NA  Age  Age, age of menarche, age of first delivery, number of full-term pregnacies, body mass index  NA  NA  Age, ages of menarche  Age, ages of menarche  Age, ages of menarche  NA  Age, gender  NA  NA  NA  Constraints  Age, gender |
| Total | 2058/3026 4131/5387 | | | | | | | | | |

M, Male; F, Female; NA, Not available; HWE, Hardy-Weinberg equilibrium.
