# Supplementary material for: Hypoxia-inducible factor-1α gene polymorphisms and cancer risk: a meta-analysis
Source: J Exp Clin Cancer Res. 2009 Dec 27;28(1):159. doi: 10.1186/1756-9966-28-159 (PMC2804603; doi:10.1186/1756-9966-28-159)
Supplement: Additional file 3 — Genotype and allele distribution of hypoxia- inducible factor -1α 1772 C/T and 1790 G/A polymorphisms of individual studies included in the meta-analysis. [file 1756-9966-28-159-S3.DOC]

**Additional file 3: Genotype and allele distribution of *hypoxia- inducible factor -1α* 1772 C/T and 1790 G/A polymorphisms of individual studies included in the meta-analysis.**

| Study | Cases (1790 G/A) | | | | | Controls (1790 G/A) | | | | | Cases (1772 C/T) | | | | | Controls (1772 C/T) | | | | |
| --- | --- | --- | --- | --- | --- | --- | --- | --- | --- | --- | --- | --- | --- | --- | --- | --- | --- | --- | --- | --- |
|  | GG | GA | AA | G | A | GG | GA | AA | G | A | CC | CT | TT | C | T | CC | CT | TT | C | T |
| Foley (9)  Li (10)  Orr-Urtreger (11)  Chau (12)  Lee (13)  Apaydin (14)  Kim (15)  Horree (16)  Konac (17)  Endometrial cancer  Ovarian cancer  Cervical cancer  Fransen (18)  Kuwai (19)  Ollerenshaw (20)  Clifford (21)  Munoz-Guerra (8)  Ling (22)  Tanimoto (6) | -  1053  198  -  -  102  87  -  21  47  32  189  -  65  35  40  -  51 | -  13  2  -  -  0  3  -  0  2  0  9  -  67  0  21  -  4 | -  0  0  -  -  0  0  -  0  0  0  0  -  14  0  3  -  0 | -  2119  398  -  -  204  177  -  42  96  64  387  -  197  70  101  -  106 | -  13  2  -  -  0  3  -  0  2  0  9  -  95  0  27  -  4 | -  1247  298  -  -  98  94  -  107  107  107  247  -  239  140  130  -  101 | -  17  2  -  -  4  7  -  0  0  0  9  -  39  4  9  -  9 | -  0  0  -  -  0  1  -  0  0  0  0  -  10  0  0  -  0 | -  2511  598  -  -  200  195  -  214  214  214  503  -  517  284  269  -  211 | -  17  2  -  -  4  9  -  0  0  0  9  -  59  4  9  -  9 | 65  818  287  161  1207  79  81  50  4  34  10  167  100  16  30  57  84  45 | 30  209  99  29  119  21  8  5  12  14  14  28  0  54  5  6  11  10 | 0  14  16  6  6  2  1  3  5  1  8  3  0  90  0  7  0  0 | 160  1845  673  351  2533  179  170  105  20  82  34  362  200  86  65  120  179  100 | 30  237  131  41  131  25  10  11  22  16  30  34  0  234  5  20  11  10 | 175  995  217  179  1245  68  93  463  68  68  68  213  89  1  110  106  93  98 | 13  221  80  14  123  29  9  84  37  37  37  43  11  90  27  25  11  12 | 0  18  3  3  1  5  0  12  2  2  2  2  0  71  6  8  0  0 | 363  2211  514  372  2613  165  195  1010  173  173  173  469  189  92  247  237  197  208 | 13  257  86  20  125  39  9  108  41  41  41  47  11  232  39  41  11  12 |
| Total | 1920 | 121 | 17 | 3961 | 155 | 2915 | 100 | 11 | 5930 | 122 | 3295 | 674 | 162 | 7264 | 998 | 4349 | 903 | 135 | 9601 | 1173 |
| Prevalence (%) | 93.3 | 5.9 | 0.8 | 96.2 | 3.8 | 96.3 | 3.3 | 0.4 | 98.0 | 2.0 | 79.8 | 16.3 | 3.9 | 87.9 | 12.1 | 80.7 | 16.8 | 2.5 | 89.1 | 10.9 |
